# Pulsed Electric Field and Freeze-Thawing Pretreatments for Sugar Uptake Modulation during Osmotic Dehydration of Mango

**DOI:** 10.3390/foods11172551

**Published:** 2022-08-23

**Authors:** Pingdwendé Assana Zongo, Seddik Khalloufi, Sergey Mikhaylin, Cristina Ratti

**Affiliations:** 1 Food Science Department, Institute of Nutrition and Functional Foods, Université Laval, Quebec City, QC G1V 0A6, Canada; 2 Soils and Agri-Food Engineering Department, Institute of Nutrition and Functional Foods, Université Laval, Quebec City, QC G1V 0A6, Canada

**Keywords:** osmotic dehydration, mango, sugar uptake, pretreatments, freeze-thawing, pulsed electric field

## Abstract

Osmotic dehydration kinetics depends on food tissue microstructure; thus, modulation of mango porosity could help selectively enhance water removal over sugar gain. In this present study, pretreatments of freeze-thawing (freezing at −36 °C for 2 weeks and thawing at 4 °C for 24 h) and pulsed electric field (1 kV/cm, 10 and 30 pulse numbers), were applied to mango 1 cm-thickness slices prior to osmotic dehydration conducted at 40 °C for 4 h. Three different 60 °Brix agave syrup solutions with or without added polysaccharides (inulin or xanthan gum) were used in the osmotic dehydration operation. Water loss (*WL*), sugar gain (*SG*) and microstructure images were used to compare the effects of pretreatments on mango osmotic dehydration efficiency. Results indicated that pulsed electric field (PEF) pretreatment increased slightly *WL* during osmotic dehydration, contrary to freeze-thawing (F-T), which for most cases led to a decrease. As for solids uptake, due to higher damage induced by F-T to mango tissue, *SG* was higher than for fresh and PEF pretreated mangoes. Using xanthan gum as additive to agave syrup solution, helped to decrease sugar uptake in frozen-thawed mango due to an increase in solution viscosity. A similar *WL/SG* ratio was obtained with frozen-thawed mango in solution with xanthan gum. Therefore, in the case of frozen-thawed mango, it is recommended to use an osmotic solution with high viscosity to obtain low sugar uptake in the final product. The novelty of this contribution is twofold: (i) using pretreatments (F-T or PEF) to minimize sugar uptake during osmotic dehydration, and (ii) using agave syrup with added polysaccharides to enrich final product with inulin.

## 1. Introduction

Consumers are encouraged to include more fruits and vegetables in their diet [1]. Processed fruits (semi-dried, dried, juice, purees) include a wide range and are available throughout the year, unlike fresh fruits which are seasonal, such as in the case of mango. Classified in the tropical fruit category, mango is available for a short period of time. Its taste, flavor and nutrients (vitamins B1, B2, C, A, antioxidant beta-carotene) contribute to its success with consumers worldwide [2]. Drying is the most common method used to prolong mango shelf life and additionally contributes to the economy in tropical countries where mango grows most commonly. Methods of mango drying consist of conventional drying (air drying) or non-conventional drying such as osmotic dehydration.

Due to the high temperature used in air-drying technology (often above 70 °C), oxidation and Maillard reactions may occur leading to degradation of beneficial components such as polyphenols, pigments, vitamins, etc. [3,4]. Osmotic dehydration, which consists of the partial removal of water through the immersion of a cellular food in a hypertonic solution (sugars are the most used osmotic solute), is recognized as a minimal processing technology due to the medium or low temperature used [5]. Osmotic dehydration is a simultaneous countercurrent mass transfer process, where water is lost, and sugar is gained. Osmotic dehydration’s main purpose is to decrease water activity to preserve fruits or vegetables for longer periods. In addition, it is a low energy process technology which produces high quality products due to low temperatures and absence of oxygen which restrains enzymes responsible for browning during processing [6]. Osmotically dehydrated products are reported to have better color, flavor, taste, texture and nutrients retention, close to those of the fresh product [6]. It is also possible to modulate food chemical composition by incorporating high quality nutrients into the final product [7]. However, major drawbacks of osmotic dehydration are the uptake of sugar and slowness of water loss. Nowadays, consumers tend to reduce sugar intake in their diet, so increased sugar content in dehydrated mangoes may negatively impact their commercial attribute and limit consumption. Many factors influence osmotic dehydration kinetics and can be optimized for sugar uptake reduction. Microstructure of the tissue shows variation in pore distribution and interconnectivity within the fruit matrix which affects the pathways for mass transfer in osmotic dehydration [6]. Water loss and solute uptake increase as time, temperature, or solution concentration increase, while an increase in solute molecular weight decreased solute uptake. High temperature favors mass transfer but may lead to undesirable changes to the plant material at temperatures above 50 °C in terms of color, flavor, aroma and nutrients degradation, in addition to enhancing sugar gain which nowadays is unfavored by consumers [8]. The treatment time can be reduced depending on conditions such as concentration, temperature of the osmotic solution, and pre-treatments (pulsed electric field, ultrasound, high hydrostatic pressure, etc.). The rate of osmotic dehydration increases with the solution concentration because osmotic pressure and chemical potential are proportional to concentration [9]. Several methods have been proposed to address sugar uptake issue in osmotic dehydration, from using centrifugal force [10,11] or high molecular solute such as corn syrup solids [12] to coating [13] and high viscosity solutions [14]. More recently, pretreatments that modify cell structure distribution of the material to be dehydrated were explored to modulate osmotic dehydration kinetics in terms of favoring water loss, with minimal additive uptake. Modification of cell microstructure leads to removal of barriers in mass transfer, such as cell wall integrity and entrapped gas in the pores, and additionally increases porosity [9,15]. Pretreatments such as freeze-thawing (F-T) and pulsed electric field (PEF) are well known for their direct effect on material microstructure and have been used to enhance drying kinetics [16,17]. In many countries, freezing is necessary to keep mangoes for longer periods due to short production seasons throughout the year, and thus mangoes are often frozen-thawed prior to osmotic dehydration. The first step in F-T process is freezing, during which ice formation modifies the tissue structure by depolymerization of cell walls, cell membrane breakage and osmotic pressure alteration [18], in addition to degassing [9]. The second step is thawing, which leads to softening of the tissue through ice melting and drip loss [18]. Due to these physicochemical modifications induced to the tissue, F-T has been successfully used to enhance air drying of apple, eggplant and beetroot [19], blueberries [20], okra [21] and garlic [22]. In the case of osmotic dehydration, rarer use of F-T as a pretreatment has been reported, but it has been used in the case of apples [23]. This study showed increasing effect of F-T pretreatment on sugar gain. PEF is the application of short repeated high voltage pulses to a biological tissue [24]. During PEF application, when the electrical potential difference of the cell’s membrane, also known as transmembrane potential, reaches a threshold value of 0.2–1.5 V [25,26], it can induce a temporarily loss of membrane semi-permeability called electro-permeabilization [25] and the openings in cell membranes through pore formation or expanding of initial pore size, known as electroporation [27,28]. The PEF treatment outcome is related to field strength (kV/cm), pulse number, energy, frequency and total treatment time [29,30]. An estimation of cell permeabilization induced by PEF can be made through the disintegration index, Zp. Tedjo et al. [31] reported that an increase in Zp in mango tissue (cv. Kent) after PEF treatment is proportional to the increase of field strength and pulse numbers. Increases in water loss and solids uptake after osmotic dehydration of PEF treated mango and apples were found in osmotic dehydration studies from [31,32] respectively. Some authors, though, have observed lower sugar uptake after PEF pretreatment, such as the work on osmotic dehydration of kiwifruit in a 61.5 °Brix sucrose solution [33]. Therefore, it could be possible to optimize the conditions of the PEF pretreatment to modulate tissue microstructure in order to favor water loss over sugar uptake.

Thus, the present study explores the impact of pretreatments such as F-T and PEF on mango slices so as to modify the fruit tissue microstructure before osmotic dehydration with the aim of obtaining higher water loss with minimal sugar uptake.

## 2. Materials and Methods

### 2.1. Mango Samples’ Preparation for Experiments

Fresh Tommy Atkins mangoes were purchased in a local supermarket and kept at ambient temperature (20 °C) for 4–5 days before further processing. Firmness was measured with a texturometer EZ-test (Shimadzu, Kyoto, Japan), as explained in Section 2.1.1, to select mangoes for the experiment. Then, mangoes were washed, rinsed, peeled, and cut manually into cuboid slices of 2.5 cm width, 5 cm length and 1 cm thickness. The samples weighed approximatively 12 g ± 2. Random samples were then selected for further physicochemical analysis.

#### 2.1.1. Firmness Analysis

Firmness of fresh samples (5 replicates) were measured with an EZ-test texturometer (Shimadzu, Kyoto, Japan) following a slightly modified protocol from [31]. A cuboid shaped mango sample was placed between a flat probe and a flat platform, both separated by a 10 mm distance. Firmness was recorded as the maximum force in Newtons (N) required to compress the sample to a depth of 5 mm on the platform with a speed of 1 mm/s.

#### 2.1.2. Titratable Acidity, pH and Soluble Solids

Mango flesh was homogenized in a blender (Hamilton Beach, Markham, ON, Canada) to obtain a puree. Fifty (50) mL of puree was used to measure the pH with an automatic titrator Orion T910 (Thermofischer scientific, Ottawa, ON, Canada). For titratable acidity measurements, 40 mL of water was added to 10 mL of puree into a beaker and homogenized. Then, titration was done in an automatic titrator Orion T910 with 0.1 N NaOH solution until point of neutrality [34]. Triplicates were made for each analysis. Results were reported as percentage of citric acid:

(1) $$\mathrm{Percentage}\;\mathrm{citric}\;\mathrm{acid}=\frac{\mathrm{Titer}\;\times 0.0064\times 100}{10\;\left(\mathrm{mL}\;\mathrm{juice}\right)}\;$$

where the factor for citric acid was 0.0064 [35].

Total soluble solids (°Brix) was measured in mango puree with a refractometer Atago (PAL-1, Tokyo, Japan).

### 2.2. Pre-Treatments

#### 2.2.1. Freeze-Thawing

Mango cuboid samples were frozen at −36 °C in a Sanyo medical freezer (MDF 235, Gunma, Japan) for 2 weeks, then thawed at 4 °C in a refrigerator for 24 h before osmotic dehydration. F-T was used as a control for total tissue destruction for comparison purposes with PEF pre-treatment. Please note that fresh mangoes were cut in the same cuboid shape on the day of the osmotic dehydration experiment to be used as control for F-T and PEF.

#### 2.2.2. Pulsed Electric Field

PEF treatment was carried out in a PEF-Cellcrack III batch system (DIL, Quakenbrück, Germany) with output voltage up to 30 kV, and frequency of 2 Hz. The treatment chamber consisted of two parallel stainless electrodes separated by 300 mm distance. Two liters of tap water (~0.2 mS/cm) was added to the treatment chamber and served as a conductor between the electrodes.

Cuboid mango slices (2.5 cm × 5 cm, 1 cm thick) were first weighed and then about 420 g were added to the water in the treatment chamber. Pre-treatment was made at 1 kV/cm field strength, and number of pulses applied were 10 and 30. After each treatment, mango slices were gently blotted onto a paper to remove superficial water.

Prior to osmotic dehydration, pretreated samples were tested to estimate the tissue damage provoked by PEF treatments through disintegration indexes based on firmness or electrical conductivity (please refer to details in the following sections). Control was prepared according to [36,37] by freezing samples at −36 °C for 24 h followed by thawing at 21 °C for 24 h. Samples were then kept at ambient temperature before firmness and electrical conductivity measurements.

Membrane permeabilization is induced during PEF through formation of pores. To measure the extent of permeabilization, a disintegration index *Z* is the most common indicator since its value increases with the pretreatment intensification (PEF strength, pulses number, pulse width and duration). The disintegration index *Z* ranges from 0 (intact membrane) to 1 (totally disintegrated membrane) [38]. Two methods were used for the disintegration index determination. The firmness method and the electrical conductivity method were used to assess the change in mango tissue conductivity due to PEF treatment.

#### 2.2.3. Tissue Disintegration Evaluation

##### Firmness Method

Firmness (F) of fresh, frozen-thawed (used as control as described in the previous paragraph) and PEF treated samples was measured with an EZ-texturometer as described in (Section 2.1.1). At least, 5 samples were measured for each treatment at ambient temperature. Disintegration index of the tissue based on firmness (*Z_F_*) was then estimated with the following equation [39]:

(2) $$Z_{F}=\frac{F_{i}-F_{t}}{F_{i}-F_{d}}\;$$

where *F_i_*, *F_d_* and *F_t_* are the firmness (N) of fresh, F-T and PEF treated mango samples, respectively.

##### Electrical Conductivity Method

Electrical conductivity (σ) was obtained indirectly through electrical resistance measurements by using a multimeter (Mastercraft model 052-0052-2, Toronto, ON, Canada) connected to two lab-made plate copper electrodes (2.5 cm × 5 cm, 1 mm thick) between which a cuboid shaped mango sample (fresh, F-T or PEF treated) was placed. At least 5 samples were tested for each type. The equation below was used to calculate the electrical conductivity according to [40]:

(3) $$\sigma =\frac{E}{R\times S}\;$$

where σ is electrical conductivity (S/m), *E* is sample thickness (m); *R* is the electrical resistance (Ω) and *S* represents the surface of the electrode (m^2^). Then, the disintegration index of the tissue (*Z_σ_*) based on electrical conductivity was estimated as in [38]:

(4) $$Z_{\sigma }=\frac{\sigma_{i}-{\;\sigma }_{t}}{\sigma_{d}-\;\sigma i}\;$$

where σ*i*, σ*d* and σ*t* are the values of electrical conductivity (S/m) for initial, for F-T and PEF treated mango for frozen-thawed and PEF treated mango samples, respectively.

### 2.3. Osmotic Solutions

Osmotic solutions used for this study were based on a ‘lab-made’ model of agave syrup (AS) to which inulin or inulin + xanthan gum was added. Agave syrup (AS) consisted of a mix of simple sugars to mimic proportions in dry weight in real agave syrup (79% fructose, 20% glucose, and 1% sucrose) in distilled water at 60 °Brix. Then, 5% inulin or (0.3% xanthan gum + 5% inulin) were added to obtain two other osmotic solutions with presence of long-chain polysaccharides (these solutions were labelled as AS + 5%I and AS + 0.3%XG + 5%I, respectively). The concentrations of inulin and xanthan gum were chosen for effective sugar reduction based on previous published results [14]. An Atago Pocket refractometer PAL-2 (Tokyo, Japan) was used to measure soluble solids content (°Brix) of the solutions.

### 2.4. Osmotic Dehydration

Osmotic solution was heated up to 40 °C in a water bath (Fischer scientific, model Isotemp 1016 S, Pittsburgh, PA, USA), before immersing mango samples, which were identified in individual cages. The solution to sample ratio was 1:100 (*w:w*) to avoid dilution. Osmotic dehydration was carried out for up to 4 h. F-T samples and PEF-treated samples were osmotically dehydrated in two separate experiments and each experiment had its own control (non-treated samples). A sample was taken out of the solution every 1 h, rinsed quickly and blotted with paper, then weighed.

Afterwards, osmotic dehydrated samples were lyophilized at 20 °C, 30 millitorr vacuum for 72 h. Freeze-dried samples were then weighed (Mettler Toledo AB104-S, Greifensee, Switzerland) to obtain their dry mass (*M_d_*). Solids Gain (*SG*) and Water Loss (*WL*) represent, respectively, the water removed and the solids uptake from the mango samples after osmotic dehydration, based on initial mass of mango samples:

(5) $$WL\;\left(\%\right)=100\times \frac{\left(P_{O}-M_{dO}\right)-\left(P-M_{d}\right)}{P_{0}}\;$$

(6) $$SG\;\left(\%\right)=100\times \frac{M_{d}-M_{dO}}{P_{O}}\;$$

where *M_d0_* is the initial dry matter (g); *M_d_*, final dry matter (g); *P_0_*, initial sample mass (g), and *P*, the final sample mass (g). For water loss, unit was g of water/100 g fresh mango and, for sugar gain, g of sugar/100 g of fresh mango. Additionally, osmotic dehydration efficiency (*ODE*) was estimated as the ratio of water loss to sugar gain at equilibrium:

(7) $$ODE=\frac{WLeq}{SGeq}\;$$

*ODE* was expressed in g water lost/g solids gained and *WLeq* (g of water/100 g fresh mango) and *SGeq* (g of sugar/100 g of fresh mango) represent, respectively, water loss and sugar at equilibrium.

### 2.5. Microscopic Analysis

Fresh, frozen-thawed and PEF-treated mango samples before and after osmotic dehydration were individually immersed in classic plant fixator (FAA) [41], a mixture of 10 mL of formaldehyde 37%, 35 mL distilled water, 5 mL of glacial acetic acid and 50 mL of alcohol 99%. Before microscopic observation, the samples were taken out of the FAA and cut with a slicer into 1-cm cubes. The microscopic images were taken at the mango slice surface by confocal method [42] with a Leica SP8 microscope (Ontario, ON, Canada). Image J software program (version 2.1.0/1.53c, Java 1.8.0, National Institutes of Health, Bethesda, MD, USA) was used for images extracting and scaling at 100 μm [43].

### 2.6. Statistical Analysis

Experiments were made in triplicate, except for firmness (5 replications). The statistics analysis was carried on with Rstudio software (RStudio-1.2.5033, Integrated Development for R. RStudio, Inc., Boston, MA, USA). Data were subjected to ANOVA (analysis of variance) and means were compared with Tukey test. The confidence level used was 95%.

## 3. Results and Discussion

### 3.1. Mango Physico-Chemical Characteristics

Table 1 shows the physico-chemical characteristics of mangoes used for osmotic dehydration experiments before F-T and PEF pretreatments. Average values of pH, titratable acidity, soluble solids, moisture content, and firmness are reported. Average pH value of fresh mango was 3.86 ± 0.37. Mango is considered an acidic fruit due to its content in citric and malic acids, with pH generally lower than 6. Values of pH in literature for ripened Tommy Atkins mango ranged from 3.2 to 4.5 [44,45,46]. The values reported in this study for pH are therefore in accordance with literature. Furthermore, titratable acidity which is correlated to pH, was 0.57 ± 0.07 g citric acid/100 g fresh mango which is close to value reported by [47] for cv. Kent (0.58 ± 0.02). Soluble solids (12.16 ± 2.56) and moisture content (85 g water/100 g fresh mango ± 0.03) agreed with the literature. For instance, results in [47,48] presented soluble solids of 15.7 ± 0.3 and 14.98, respectively, while moisture content was stated as 84.87 ± 1.76 [47] and 87.24 ± 1.14 [31].

Average firmness value was 49.21 N ± 11.36. Compared to the literature, mango firmness used in this study was two times the value of 25.49 N reported by [47] for ripe Kent mango, while it was close to the 52.31 N firmness value reported by [46] for Tommy Atkins. Many factors influence mango ripeness: number of days since full flowering [49], geographical region, microclimatic conditions of the mango trees, long distance during transport (i.e., from Brazil to Quebec), storage condition, etc. [50]. These can lead to heterogeneous batches that may have different physico-chemical characteristics [50,51,52,53] among which firmness is predominantly impacted, consequently leading to difficulty in managing a uniform batch of mangoes. In this study, to select mangoes with uniform ripeness as accurately as possible, the parameters presented in Table 1 were all considered.

### 3.2. Effects of F-T and PEF Pretreatments on Osmotic Dehydration Kinetics

#### 3.2.1. Water Loss

**Figure 1:**
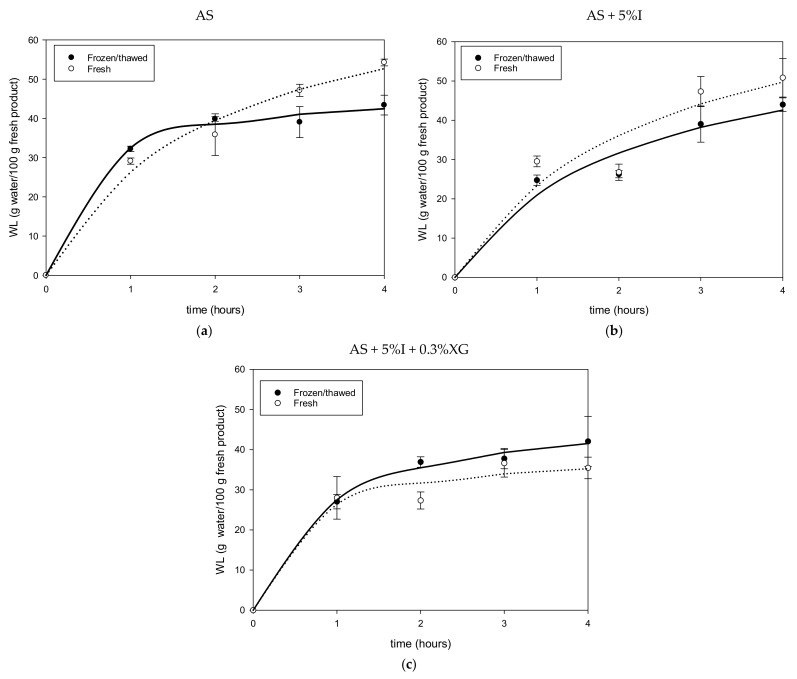
Water loss of Fresh and F-T mango during osmotic dehydration in AS (**a**), AS + %I (**b**) and AS + %I + 0.3%XG (**c**) solution.

**Figure 2:**
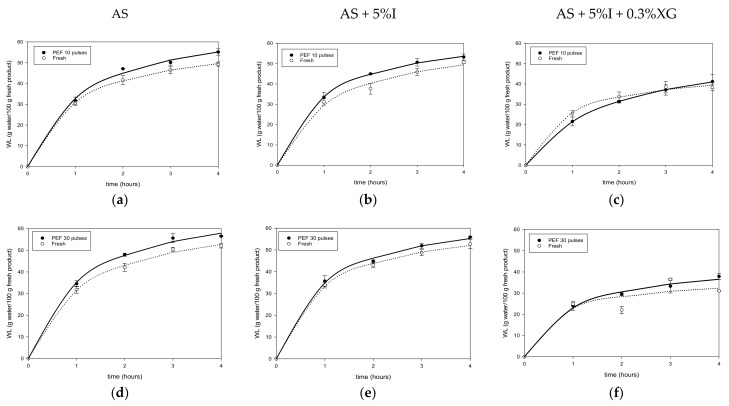
Water loss of Fresh and PEF mango during osmotic dehydration in AS (**a**,**d**), AS + %I (**b**,**e**) and AS + %I + 0.3%XG (**c**,**f**) solutions.

Figure 1a–c and Figure 2a–f illustrate results for water loss (*WL*) during osmotic dehydration of fresh, F-T and PEF treated mangoes in AS, AS + 5%I and AS + 5%I + 0.3%XG osmotic solutions.

*WL* increased with time (*p* < 0.05) during osmotic dehydration of mango. It can be observed from Figure 1a,b that F-T pretreatment decreased *WL* during osmotic dehydration of mango samples in agave syrup solutions with and without added inulin, while slightly improving it when xanthan gum was added to agave syrup/inulin solution (Figure 1c). While for PEF 10 and 30 pulses, *WL* of the samples increased slightly for the three osmotic solutions. Mango osmotically dehydrated with AS + 5%I + 0.3%XG showed lowest *WL* for fresh, F-T and PEF samples compared to AS and AS + 5%I. The different behavior reported in AS + 5%I + 0.3%XG is related to high viscosity of this solution due to addition of a thickening agent such as xanthan gum [14]. Increasing markedly, the solution viscosity may cause a change in the control for mass transfer during osmotic dehydration, from internal (solid matrix) to external (solution). Thus, a pretreatment such as F-T impacting on the cellular matrix may have less effect on *WL*. Similar results of F-T on *WL* were found for apple [23], strawberries [54], African star apples [55] and pomegranate seeds [56].

Considering the effect of PEF pulses number on mango *WL,* increasing the number of pulses from 10 to 30 pulses had a positive effect only for AS + 5%I. PEF could improve cell permeabilization by creating pores on the tissue surface through electro plasmolysis [57]. These new pores could be used as supplementary pathways for water transport out of the tissue during dehydration, particularly in osmotic dehydration. Prior studies have already reported similar results on *WL* increment after PEF treatment of mango and other fruits. A previous study on osmotic dehydration of mango in a sucrose 50 °Brix solution after PEF treatment was conducted [31], where they reported a slight increase in water loss after PEF pretreatment compared to untreated mango. Similar *WL* increase was reported by [58] for kiwifruit, [59] for carrots, and [60] for apples. Contrary to expectations, no significant difference was found between 10 and 30 pulses treatment on mango *WL* with AS and AS + 5%I + 0.3%XG osmotic solutions, although the energy applied to mango samples increased with number of pulses (4 and 13.5 kJ/kg for 10 and 30 pulses, respectively). In the literature, there are no consistent reports on the correlation between PEF pulse number and *WL* increase during osmotic dehydration. For instance, authors in [61] found that *WL* of osmotically dehydrated apples in sucrose 60 °Brix solution increased as PEF pulses increased from 10 to 50 at 5 and 10 kV/cm field strength. Similarly, Ade-Omowaye et al. [62] succeeded in improving *WL* of PEF treated bell peppers. On the other hand, pulse number increment presented no correlation in improving *WL* in apples [63] indicating that PEF effect may depend on the fruit type. Due to their different impact on the tissue, PEF treatment showed positive impact on *WL*, unlike F-T which led to a decrease.

#### 3.2.2. Solids Gain

**Figure 3:**
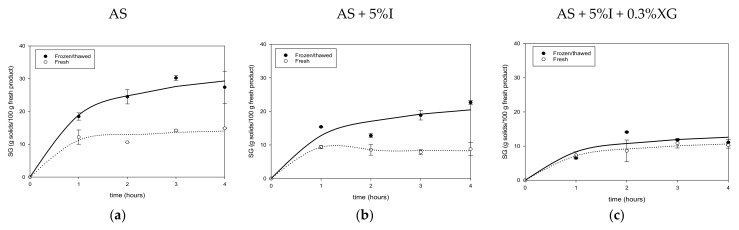
Solids gain of Fresh and F-T mango during osmotic dehydration in AS (**a**), AS + %I (**b**) and AS + %I + 0.3%XG (**c**) solutions.

**Figure 4:**
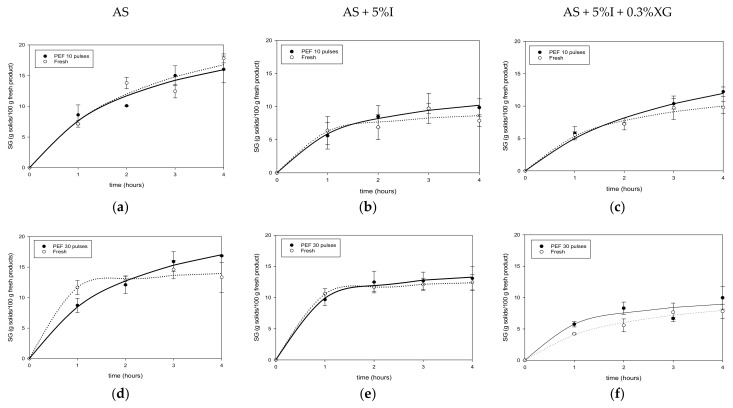
Solids gain of Fresh and PEF mango during osmotic dehydration in AS (**a**,**d**), AS + %I (**b**,**e**) and AS + %I + 0.3%XG (**c**,**f**) solutions.

Figure 3a–c and Figure 4a–f illustrate solids gain evolution in F-T and PEF mango and their respective controls (fresh).

Solids gain (*SG*) increased with time and reached the highest values at 4 h. For F-T mango, a two-fold increase in solids was observed compared to fresh in AS and AS + 5%I solutions, except for AS + 5%I + 0.3%XG solution, for which the increase was just 1.09%. Final values of solids were 14.88%, 8.74%, 10.84% for fresh mango and 27.42%, 22.68%, 11.08% for F-T ones in AS, AS + 5%I and AS + 0.3%XG + 5%I osmotic solutions, respectively (Figure 3a–c). As can be seen, solids gain remains similar for both fresh and F-T mango in AS + 5%I + 0.3%XG. Regarding PEF pretreatment (Figure 4a–f), compared to fresh mango, PEF slightly increased *SG* at 10 pulses for AS + 5%I and AS + 5%I + 0.3XG, while it increased *SG* for all three osmotic solutions after PEF 30 pulses pretreatment. However, increasing pulses number from 10 to 30 did not show a significant effect on *SG* improvement. In addition, there was a variable effect of pulse numbers according to the osmotic solution.

In comparison to fresh and PEF mango, F-T pretreatment prompted higher solids gain after osmotic dehydration. This could be related to the type of damage induced on the tissue. F-T can result in both physical (cell rupture by ice crystal growth) and chemical damages (biochemical reactions after cell fracture of mango tissue), destroying the cellular compartments through pectin hydrolysis, leading to cell separation and rupture [18,64,65,66]. After thawing, melted ice crystals left spaces or voids that can be used by sugar molecules to enter easily into the tissue [67], and thus a greater flux of solids enter the matrix, as shown for *SG* in Figure 3a–c). During osmotic dehydration, *WL* is a simultaneous countercurrent flow to *SG* and has been pointed out to be controlled by diffusion [23]. Thus, greater solids flux entering the matrix due to F-T pretreatment may accumulate near the surface and act as a barrier for *WL*. In addition, cell walls collapse, and the deformation of cellular network (shown later through microstructure results ) may increase the tortuosity of the tissue which is inversely proportional to water diffusion coefficients. In the case of PEF, electroporation is the phenomena responsible for tissue microstructure modification through increment of porosity [68], which may explain the observed slight increase in solids uptake. The new pores induced in the mango tissue may have different sizes as to the original ones which could favor selectively water molecules as was observed for *WL* increment in Section 3.2.1. Previous studies have shown that, after PEF treatment of apples, pore measurement indicated that PEF generated pores had smaller mean sizes than untreated ones [69]. In agreement with the present results, other authors also showed a slight increase of *SG* with PEF pretreatment in osmotically dehydrated mango [31], apples [60] and kiwifruit [58]. On the other hand, some studies found no change [61] or lower solids uptake [33] compared to fresh samples. The less destructive effect of PEF on the mango sample could explain the minor increase of *SG* compared to F-T.

As can be seen from Figure 3b,c and Figure 4b,c,e,f, addition of polysaccharide inulin or xanthan gum lowered sugar gain of mango in both F-T and PEF samples compared to pure AS solution. This could be explained, respectively, by the high molecular weight of inulin, and by the increase of solution viscosity for xanthan gum [14]. The high viscosity caused by xanthan gum when added to agave syrup/inulin solution could be of hindrance of the solute movement due to negative impact of viscosity on solution diffusion coefficients [14]. Inulin also reduced solids gain due to formation of a layer on the surface which creates an external resistance to solids uptake. Results on use of natural syrup to reduce sugar uptake are scarce in the literature, as most studies focused on the organoleptic and nutrients properties of the final product as in the case of sugar beet molasses [70], maple syrup [71] or honey [72]. However, some authors have shown similar effect of high viscosity and polysaccharide content with corn syrup solids solution for osmotic dehydration of papaya [73] and mango [14].

### 3.3. Effects of F-T and PEF Pretreatments on Equilibrium Values during Osmotic Dehydration

Table 2 and Table 3 show equilibrium *WL* and *SG* values (*WLeq* and *SGeq*) for F-T and PEF mango with their respective controls (fresh). Additionally, osmotic dehydration efficiency (*ODE)* is presented.

In terms of *WL*, Table 2 shows that *WLeq* is higher for fresh mango than for F-T mango, except for AS + 5%I + 0.3%XG solution in agreement with *WL* maximum values shown in Figure 1a–c. Higher *SGeq* (Table 2) in mangoes was obtained after F-T treatment as found previously. The *ODE* values showed that fresh mango had better efficiency than F-T pretreated mangoes and lower values of *SGeq*. In addition, F-T mangoes dehydrated in AS + 5%I + 0.3%XG solution presented an interesting *ODE* (3.61 ± 0.11) with a reasonable solids’ intake (11.57 ± 0.52 g solids/100 g fresh product), similar to fresh mango behavior. PEF pretreatment increased slightly *WLeq* and *SGeq* at 10 pulses and 30 pulses compared to fresh mango. In general, PEF did not show a significant improvement of *ODE* values (about 3 to 5 for fresh and PEF treated samples). Compared to similar *ODE* information presented in Table 2 for F-T pretreatment (about 1 to 4), PEF pretreatment presented a higher *ODE* value which remained closer to fresh mango. These results may indicate that it is not necessary to pretreat mango with PEF due to the small impact on mass transfer.

### 3.4. Effects of F-T and PEF Pretreatments on Mango Tissue

#### 3.4.1. Effect of F-T on Mango Firmness and Electrical Conductivity during Osmotic Dehydration

Table 4 shows firmness (N) and electrical conductivity (S/m) of fresh and F-T mango, together with the respective ratio of fresh and F-T values. Values of fresh mango firmness were discussed in Section 3.1. As for electrical conductivity, a low value recorded for untreated mango (0.004 S/m ± 0.001) is often reported for food if it is not pretreated in brine solution [39]. For instance, for afresh potato cylinder of 30 mm diameter, electrical conductivity was found to be less than 0.1 S/m [39] and a similar range was measured for carrot particles (6 mm–13 mm) [40]. This only increased after pretreatment, demonstrating an effect on the tissue properties.

The firmness ratio of fresh and F-T mango (4.0 ± 2.26) indicated a four-fold decrease of firmness after the F-T treatment. It is well known that firmness of fruit tissue is reduced through F-T due to cell wall breakdown and collapse [74]. As for the electrical conductivity ratio, it shows a value of 0.12 ± 0.02, indicating that F-T samples are about eight times more conductive than fresh mango. Electrical conductivity measures the ability to conduct a current, and in a food matrix it increases with the electrolytes’ leakage (ions, soluble solids) through the open pores generated during the thawing process, contributing to higher electrical conductivity values. These results strongly support softening theories for mango tissue because of F-T.

#### 3.4.2. Effect of F-T and PEF on Mango Disintegration Index

**Figure 5:**
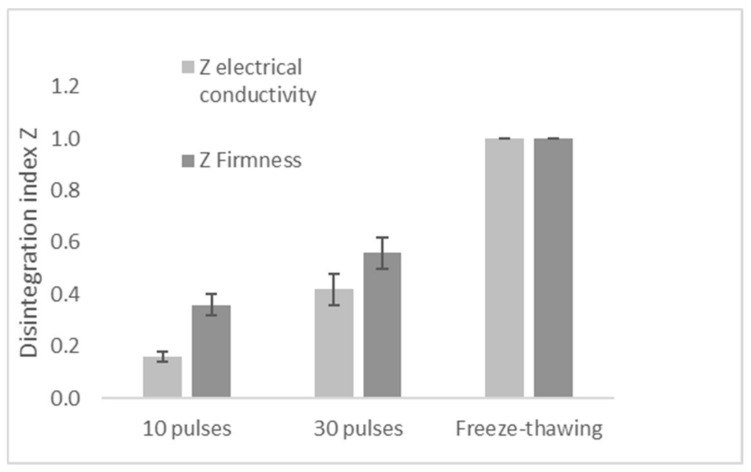
Disintegration index *Z* with electrical conductivity and Firmness methods.

Figure 5 provides results of disintegration index *Z* obtained for mango after PEF treatment of 10 pulses and 30 pulses at 1 kV/cm and 1 kHz. The maximum *Z* value (1) corresponding to F-T samples has been added to Figure 5 as a reference. In this Figure, the electrical conductivity *Z* method results are represented in light grey and firmness *Z* results, in dark grey. As can be seen from Figure 5, both methods show that disintegration index *Z* increases with PEF pulse number. The minimum *Z* values were 0.16 and 0.36 while the maximum were 0.42 and 0.56, respectively, for 10 and 30 pulses. This agrees with other studies on the effect of number of pulses on tissue disintegration index. For instance, Tedjo et al. [31] showed an increase of *Z* disintegration values of mango from 0.18 to 0.58 with the increase in number of pulses from 10 to 100. Similarly, Dermesonlouoglou et al. [75] reported an enhancement of disintegration index *Z* in Goji Berry (up to 0.38) with increasing the number of pulses (750, 1500, 7500) at 2.8 kV/cm. The tendency of *Z* values observed by both electrical conductivity and firmness methods is similar, though the firmness method gave higher values of *Z* than with the electrical conductivity method. As non-destructive methods are becoming a trend in evaluating food material properties in the food industry, the electrical method is promising because it keeps the integrity of the product, does not necessitate high-cost material and is simple to use than the texturometer for firmness evaluation. However, this lab-made tool that has been developed for the mango electrical conductivity analysis in this study is still in the experimental stage and further research is needed to improve the technology for more accuracy.

The modification of the disintegration index observed through the results of the present study indicated that PEF treatment improved permeabilization of mango tissue. This confirms that formation of pores happened after PEF pretreatment facilitating water loss and solids gain (Figure 3 and Figure 4). The totally destroyed cellular structure caused by F-T (*Z* = 1), drastically reduces internal barrier for mass transfer, allowing more solids uptake due to loss in selective permeability. PEF treated sample, on the other hand, induces minor modifications of cellular tissue creating new paths for loss of small molecules of water along with solids uptake.

### 3.5. Effects of F-T and PEF Pretreatments on Mango Microstructure during Osmotic Dehydration

**Figure 6:**
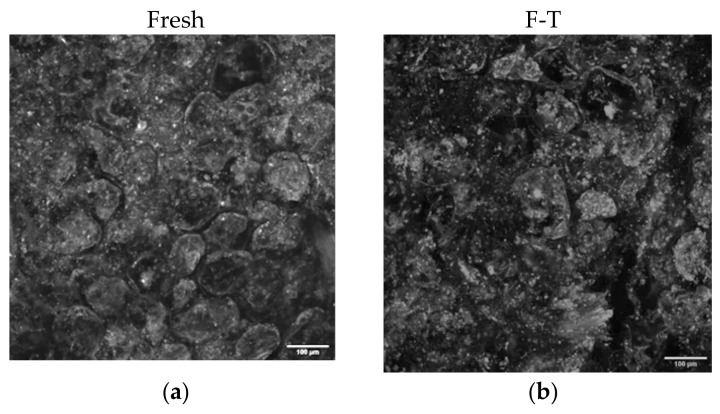

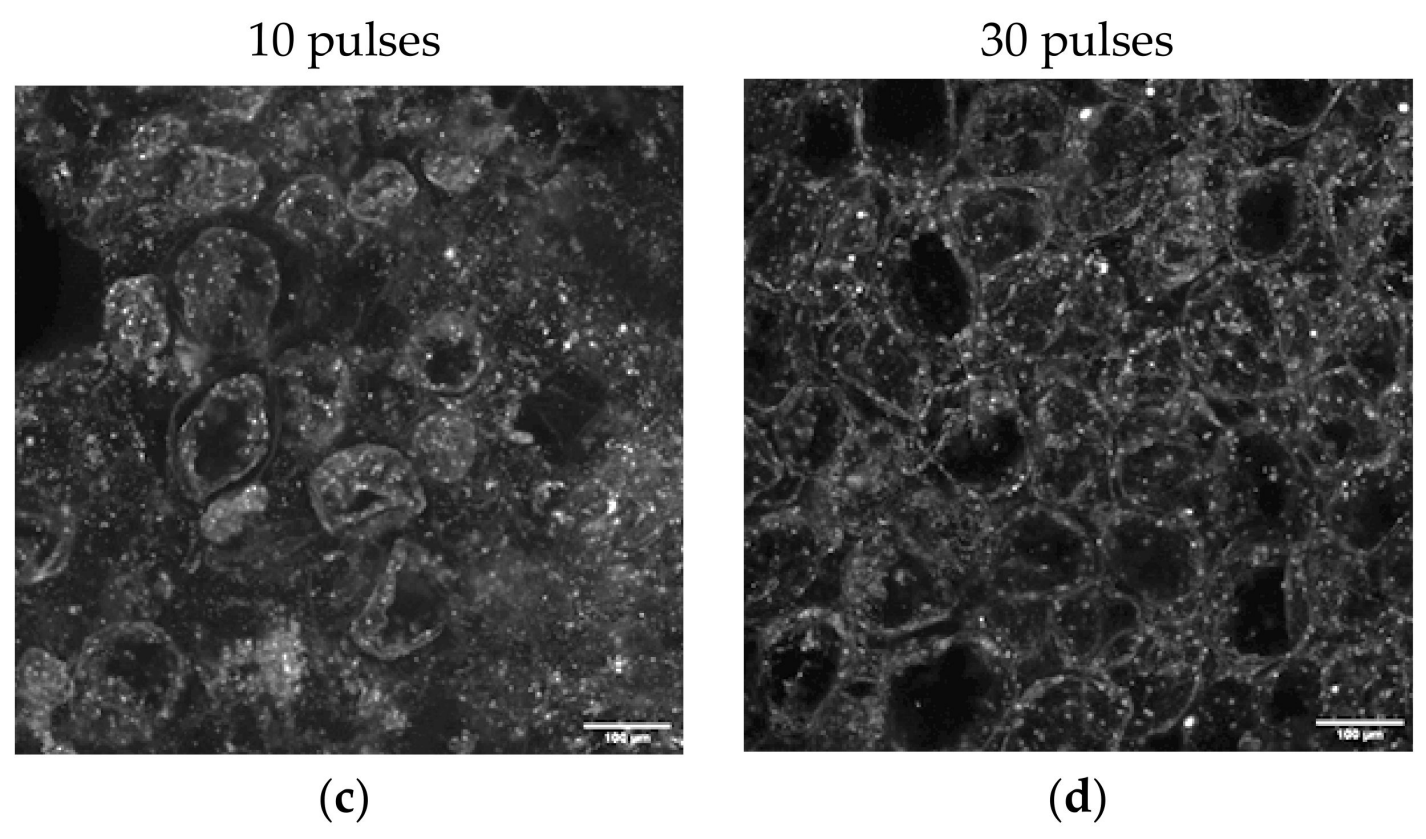
Microstructures of non-osmotic treated mango: fresh mango (**a**); after F-T (**b**); after 10 pulses (**c**) after 30 pulses (**d**). The white scale bar represents 100 μm.

Figure 6 illustrates surface microstructure of fresh (Figure 6a), F-T (Figure 6b) and PEF treated mango (Figure 6c,d for PEF10 and PEF30, respectively) before osmotic dehydration.

Figure 6a shows in fresh mango a dense distribution of oval cells, each surrounded by a cellular membrane. A thin space marking the separation between different intact cells can also be observed. After F-T pretreatment (Figure 6b), larger spaces and structural collapse are perceived throughout. Additionally, multiple puncture holes all over the tissue are noticeable. According to [76] these holes are attributed to spaces previously occupied by ice before the thawing step. The observed damage to cell membrane disabled its capacity to act as a semipermeable barrier during movement of solutes [64] and, thus, it can explain the increase in sugar uptake of F-T mango compared to fresh (Figure 3a–c) and PEF (Figure 4a–f). The increased tortuosity caused by cell wall collapse may explain the difficulty for water in using the diffusion paths, resulting in lower *WL* (Figure 1a,b). On the contrary, cellular structure of mango treated with 10 and 30 pulses (Figure 6c,d) showed oval and round cells which still had intact cell wall and cell membrane as in the fresh mangoes (Figure 6a). These images indicate that electroporation phenomenon preserves cell structure better than F-T treatment (Figure 6b). As PEF treatment increased (from 10 pulses to 30 pulses), cells appear to be closer to each other with more porosity. This may be a consequence of water leakage during electro plasmolysis, leading to pores formation which is beneficial for water removal out of the cells, leading to cell shrinkage. Then, cells separate from each other with formation of small voids.

## 4. Conclusions

This present study focused on reducing sugar uptake in mango by applying pretreatments of F-T or PEF before osmotic dehydration. F-T pretreatment was not effective in reducing sugar uptake and did not increase *WL*. However, addition of xanthan gum to agave syrup solution helped to lower sugar uptake of the final product. Therefore, in the case of fresh mango shortage, industry can use agave syrup solution with xanthan gum to produce low calorie mango. PEF pretreatment, slightly increased *WL,* and *SG*. Microscopic images have shown that F-T creates more damage on the tissue than PEF. The smaller pores induced by PEF pretreatment may be the reason for enhanced water removal compared to F-T. However, due to the short availability of fresh mango, F-T can often be necessary during off-season. In such case, using thickening agents such as xanthan gum to enrich the osmotic solution is a good alternative to lower sugar uptake during osmotic dehydration of mango.

## Figures and Tables

**Table 1:** Characteristics of mango for experimentation.

Parameters	Values
Total soluble solids (°Brix)	12.16 ± 2.56
Titratable acidity (g citric acid/100 g fresh mango)	0.57 ± 0.07
pH	3.86 ± 0.37
Moisture (g water/100 g fresh mango)	85.00 ± 0.03
Firmness (N)	49.21 ± 11.36

**Table 2:** Equilibrium and efficiency ratio of fresh and frozen-thawed mango during osmotic dehydration.

Pretreatment	*WLeq* (g Water/100 g Fresh Product)			*SGeq* (g Solids/100 g Fresh Product)			*ODE* (g Water Lost/g Solids Gained)		
	AS	AS + 5%I	AS + 5%I + 0.3%XG	AS	AS + 5%I	AS + 5%I + 0.3%XG	AS	AS + 5%I	AS + 5%I + 0.3%XG
Fresh	50.70 ^a^* ± 0.35	49.08 ^a^ ± 3.47	35.67 ^a^ ± 0.37	14.61 ^b^ ± 0.63	9.92 ^b^ ± 2.77	10.55 ^a^ ± 1.13	3.46 ^a^ ± 0.05	4.57 ^a^ ± 0.63	3.42 ^a^ ± 0.33
Frozen-Thawed	41.23 ^b^ ± 3.23	42.30 ^b^ ± 1.00	41.07 ^a^ ± 0.71	29.50 ^a^ ± 3.42	20.63 ^a^ ± 2.40	11.57 ^a^ ± 0.52	1.51 ^b^ ± 0.10	2.13 ^b^ ± 0.31	3.61 ^a^ ± 0.11

* Within the same category of variables (*WLeq*, *SGeq* or *ODE*), mean values in same column with different letters are significantly different (*p* < 0.05).

**Table 3:** Equilibrium and efficiency ratio of fresh and PEF treated mango during osmotic dehydration.

Treatment	*WLeq* (g Water/100 g Fresh Product)			*SGeq* (g Solids/100 g Fresh Product)			*ODE* (g Water Lost/g Solids Gained)		
	Osmotic Solutions			Osmotic Solutions			Osmotic Solutions		
	AS	AS + 5%I	AS + 5%I + 0.3%XG	AS	AS + 5%I	AS + 5%I + 0.3%XG	AS	AS + 5%I	AS + 5%I + 0.3%XG
Fresh10	47.93 ^a^* ± 1.00	48.31 ^a^ ± 1.16	38.42 ^a^ ± 0.64	14.04 ^a^ ± 1.95	8.80 ^a^ ± 1.56	9.76 ^a^ ± 0.44	3.49 ^a^ ± 0.59	5.68 ^a^ ± 1.05	3.92 ^a^ ± 0.14
PEF/10 pulses	52.63 ^a^ ± 1.38	51.97 ^a^ ± 1.61	42.00 ^a^ ± 4.82	15.51 ^a^ ± 1.89	9.79 ^a^ ± 0.84	11.30 ^a^ ± 0.72	3.44 ^a^ ± 0.39	5.33 ^a^ ± 0.31	3.63 ^a^ ± 0.26
Fresh30	50.36 ^A^ ± 1.61	50.76 ^A^ ± 1.18	32.57 ^A^ ± 4.21	13.97 ^B^ ± 1.96	12.27 ^A^ ± 0.72	8.25 ^A^ ± 1.11	3.67 ^A^ ± 0.52	4.15 ^A^ ± 0.27	3.95 ^A^ ± 1.05
PEF/30 pulses	55.09 ^A^ ± 1.12	53.90 ^A^ ± 0.71	36.07 ^A^ ± 1.68	17.05 ^A^ ± 1.23	12.89 ^A^ ± 1.64	8.61 ^A^ ± 0.78	3.37 ^A^ ± 0.30	4.25 ^A^ ± 0.53	4.15 ^A^ ± 0.20

* Fresh10 and Fresh30 are the controls (untreated mango) for PEF10 and PEF30 respectively. * Within the same category of variables (*WLeq*, *SGeq* or *ODE*), mean values in same column with different letters are significantly different (*p* < 0.05). Lower case letters (a, b) compared fresh10 and PEF/10 pulses samples. Uppercase letters (A, B) compared fresh30 and PEF/30 pulses samples.

**Table 4:** Firmness and electrical conductivity ratio of fresh and F-T mango.

	Firmness	Electrical Conductivity
Fresh	49.21 N ± 11.36	0.004 S/m ± 0.00
F-T	12.40 N ± 5.00	0.14 S/m ± 0.01
Ratio (Fresh/F-T)	4.00 ± 2.26	0.12 ± 0.02

## Data Availability

The data presented in this study are available on request from the corresponding author.

## References

[1] Sadler M.J., Gibson S., Whelan K., Ha M.-A., Lovegrove J., Higgs J. (2019). Dried fruit and public health—What does the evidence tell us?. Int. J. Food Sci. Nutr..

[2] Izli N., Izli G., Taskin O. (2017). Influence of different drying techniques on drying parameters of mango. Food Sci. Technol..

[3] Drouzas A.E., Tsami E., Saravacos G.D. (1999). Microwave/vacuum drying of model fruit gels. J. Food Eng..

[4] Sehrawat R., Nema P.K., Kaur B.P. (2018). Quality evaluation and drying characteristics of mango cubes dried using low-pressure superheated steam, vacuum and hot air drying methods. LWT.

[5] Torreggiani D. (1993). Osmotic dehydration in fruit and vegetable processing. Food Res. Int..

[6] Ahmed I., Qazi I.M., Jamal S. (2016). Developments in osmotic dehydration technique for the preservation of fruits and vegetables. Innov. Food Sci. Emerg. Technol..

[7] Sravani D., Saxena D. (2021). A Mini Review on Osmotic Dehydration of Fruits and Vegetables. Pharma Innov. J..

[8] Shi J., Xue S.J. (2008). Application and Development of Osmotic Dehydration Technology in Food Processing, in Advances in Food Dehydration.

[9] Phisut N. (2012). Factors Affecting Mass Transfer During Osmotic Dehydration of Fruits. Int. Food Res. J..

[10] Azuara E., Garcia H.S., Beristain C.I. (1996). Effect of the centrifugal force on osmotic dehydration of potatoes and apples. Food Res. Int..

[11] Barman N., Badwaik L.S. (2017). Effect of ultrasound and centrifugal force on carambola (*Averrhoa carambola* L.) slices during osmotic dehydration. Ultrason. Sonochem..

[12] Lazarides H.N., Katsanidis E., Nickolaidis A. (1995). Mass transfer kinetics during osmotic preconcentration aiming at minimal solid uptake. J. Food Eng..

[13] Matuska M., Lenart A., Lazarides H.N. (2006). On the use of edible coatings to monitor osmotic dehydration kinetics for minimal solids uptake. J. Food Eng..

[14] Zongo A.P., Khalloufi S., Ratti C. (2021). Effect of viscosity and rheological behavior on selective mass transfer during osmotic dehydration of mango slices in natural syrups. J. Food Process Eng..

[15] Liu C., Grimi N., Lebovka N., Vorobiev E. (2018). Convective air, microwave, and combined drying of potato pre-treated by pulsed electric fields. Dry. Technol..

[16] Taiwo K.A., Angersbach A., Ade-Omowaye B.I.O., Knorr D. (2001). Effects of Pretreatments on the Diffusion Kinetics and Some Quality Parameters of Osmotically Dehydrated Apple Slices. J. Agric. Food Chem..

[17] Toepfl S., Knorr D. (2006). Pulsed electric fields as a pretreatment technique in drying processes. Stewart Postharvest Rev..

[18] Li D., Zhu Z., Sun D.-W. (2018). Effects of freezing on cell structure of fresh cellular food materials: A review. Trends Food Sci. Technol..

[19] Vallespir F., Rodríguez Ó., Eim V.S., Roselló C., Simal S. (2018). Freezing pre-treatments on the intensification of the drying process of vegetables with different structures. J. Food Eng..

[20] Zielinska M., Sadowski P., Blaszczak W. (2015). Freezing/thawing and microwave-assisted drying of blueberries (*Vaccinium corymbosum* L.). LWT Food Sci. Technol..

[21] Xin X., Lei Z., Cunshan Z., Abu ElGasim A.Y., Hafida W., Haila M., Jin Z., Yanhui S. (2021). Ultrasound freeze-thawing style pretreatment to improve the efficiency of the vacuum freeze-drying of okra (*Abelmoschus esculentus* L.) Moench and the quality characteristics of the dried product. Ultrason. Sonochem..

[22] Feng Y., Tan C.P., Zhou C., ElGasim A.Y., Xu B., Sun Y., Haile M., Xu X., Yu X. (2020). Effect of freeze-thaw cycles pretreatment on the vacuum freeze-drying process and physicochemical properties of the dried garlic slices. Food Chem..

[23] Lazarides H.N., Mavroudis N.E. (1995). Freeze/thaw effects on mass transfer rates during osmotic dehydration. J. Food Sci..

[24] Gürsul I., Gueben A., Grohmann A., Knorr D. (2016). Pulsed electric fields on phenylalanine ammonia lyase activity of tomato cell culture. J. Food Eng..

[25] Vorobiev E., Lebovka N. (2008). Pulsed-Electric-Fields-Induced Effects in Plant Tissues: Fundamental Aspects and Perspectives of Applications. Electrotechnologies for Extraction from Food Plants and Biomaterials.

[26] Weaver J.C., Chizmadzhev Y.A. (1996). Theory of electroporation: A review. Bioelectrochem. Bioenerg..

[27] Asavasanti S., Asavanti S., Rustenpart W., Stroeve P., Barrett D.M. (2010). Permeabilization of Plant Tissues by Monopolar Pulsed Electric Fields: Effect of Frequency. J. Food Sci..

[28] Chauhan O.P., Shayanfar S., Topefl S. (2018). Cell Permeabilisation, Microstructure and Quality of Dehydrated Apple Slices Treated with Pulsed Electric Field During Blanching. Def. Life Sci. J..

[29] Angersbach A., Heinz V., Knorr D. (2000). Effects of pulsed electric fields on cell membranes in real food systems. Innov. Food Sci. Emerg. Technol..

[30] Vorobiev E.N., Lebovka N.I. (2008). Electrotechnologies for Extraction from Food Plants and Biomaterials.

[31] Tedjo W., Taiwo K.A., Eshtiaghi M.N., Knorr D. (2002). Comparison of pretreatment methods on water and solid diffusion kinetics of osmotically dehydrated mangos. J. Food Eng..

[32] Amami E., Vorobiev E., Kechaou N. (2006). Modelling of mass transfer during osmotic dehydration of apple tissue pre-treated by pulsed electric field. LWT.

[33] Traffano-Schiffo M.V., Tylewicz U., Castro-Giraldez M., Fito P.J., Ragni L., Dalla Rosa M. (2016). Effect of pulsed electric fields pre-treatment on mass transport during the osmotic dehydration of organic kiwifruit. Innov. Food Sci. Emerg. Technol..

[34] Jayasena V., Cameron I. (2008). Brix/acid ratio as a predictor of consumer acceptability of Crimson Seedless table grapes. J. Food Qual..

[35] AOAC (1990). Official Methods of Analysis.

[36] Grimi N., Vorobiev E., Lebovka N., Vaxelaire J. (2010). Solid–liquid expression from denaturated plant tissue: Filtration–consolidation behaviour. J. Food Eng..

[37] Wiktor A., Gondek E., Jakubczyk E., Nowacka M., Dadan M., Fijalkowska A., Witrowa-Rajchert D. (2016). Acoustic emission as a tool to assess the changes induced by pulsed electric field in apple tissue. Innov. Food Sci. Emerg. Technol..

[38] Lebovka N., Bazhal M., Vorobiev E. (2002). Estimation of characteristic damage time of food materials in pulsed-electric fields. J. Food Eng..

[39] Olivera D.F., Salvadori V.O., Marra F. (2013). Ohmic treatment of fresh foods: Effect on textural properties. Int. Food Res. J..

[40] Zareifard M.R., Ramaswamy H.S., Trigui M., Marcotte M. (2003). Ohmic heating behaviour and electrical conductivity of two-phase food systems. Innov. Food Sci. Emerg. Technol..

[41] Kim V.L.T.D. (2019). Formalin-Aceto-Alcohol (FAA) Solution for Killing, Fixing and Pickling Botanical Specimen.

[42] Loginova K., Lebovka N., Vorobiev E. (2011). Pulsed electric field assisted aqueous extraction of colorants from red beet. J. Food Eng..

[43] Koch Y., Witt Y., Lammerskitten A., Siemer C., Toepfl S. (2022). The influence of Pulsed Electric Fields (PEF) on the peeling ability of different fruits and vegetables. J. Food Eng..

[44] Dutra P.R.S., Cavalcanti L.A., de Assis J.S., Guerra N.B. (2005). Indicadores bioquímicos do desenvolvimento de manga Tommy Atkins produzidas no vale do São Francisco. Simpósio Bras. Pós. Colheita Frutos Trop..

[45] Lucena E.D., Silva A., Campelo I. Physicochemical characterization of mango (*Mangifera indica* L.), Tommy Atkins variety, at different stages of maturation. Proceedings of the Congresso Brasileiro de Ciência de Tecnologia de Alimentos.

[46] Santos D.B.D., Pereira M.E.C., Vieira E.L., de Lima M.A.C. (2008). Caracterização Físico-Química dos Estádios de Maturação da Manga ‘Tommy Atkins’ Produzida no Município de IaÇu-BA. Embrapa Semiárido-Artig. Periód. Indexado.

[47] Sulistyawati I., Dekker M., Fogliano V., Verkerk R. (2018). Osmotic dehydration of mango: Effect of vacuum impregnation, high pressure, pectin methylesterase and ripeness on quality. LWT.

[48] Maldonado-Celis M.E., Yahia E.M., Bedoya R., Landázuri P., Loango N., Aguillón J., Restrepo B., Guerrero Ospina J.C. (2019). Chemical Composition of Mango (*Mangifera indica* L.) Fruit: Nutritional and Phytochemical Compounds. Front. Plant Sci..

[49] Yahia E. (1999). Postharvest Handling of Mangoes. Agricultural Technology Utilization and Transfer Project, Giza, Egypt. https://www.researchgate.net/publication/277954571eg.

[50] Gianguzzi G., Farina V., Inglese P., Rodrigo M.G.L. (2021). Effect of Harvest Date on Mango (*Mangifera indica* L. Cultivar Osteen) Fruit’s Qualitative Development, Shelf Life and Consumer Acceptance. Agronomy.

[51] Galán S.V., Lu P. (2018). Achieving Sustainable Cultivation of Mangoes.

[52] Lalel H., Singh Z., Tan S. (2003). Distribution of Aroma Volatile Compounds in Different Parts of Mango Fruit. J. Hortic. Sci. Biotechnol..

[53] Sivakumar D., Jiang Y., Yahia E.M. (2011). Maintaining mango (*Mangifera indica* L.) fruit quality during the export chain. Food Res. Int..

[54] Taiwo K.A., Angersbach A., Knorr D. (2003). Osmotic dehydration of strawberry halves: Influence of osmotic agents and pretreatment methods on mass transfer and product characteristics. Int. J. Food Sci. Technol..

[55] Falade K.O., Adelakun T.A. (2007). Effect of pre-freezing and solutes on mass transfer during osmotic dehydration and colour of oven-dried African star apple during storage. Int. J. Food Sci. Technol..

[56] Bchir B., Besbes S., Attia H., Blecker C. (2012). Osmotic dehydration of pomegranate seeds (*Punica granatum*): Effect of freezing pre-treatment. J. Food Process Eng..

[57] Donsì F., Ferrari G., Pataro G. (2010). Applications of Pulsed Electric Field Treatments for the Enhancement of Mass Transfer from Vegetable Tissue. Food Eng. Rev..

[58] Dermesonlouoglou E., Zachariou I., Andreou V., Taoukis P.S. (2016). Effect of pulsed electric fields on mass transfer and quality of osmotically dehydrated kiwifruit. Food Bioprod. Process..

[59] Rastogi N., Eshtiaghi M., Knorr D. (1999). Accelerated Mass Transfer During Osmotic Dehydration of High Intensity Electrical Field Pulse Pretreated Carrots. J. Food Sci..

[60] Amami E., Vorobiev E., Kechaou N. (2005). Effect of Pulsed Electric Field on the Osmotic Dehydration and Mass Transfer Kinetics of Apple Tissue. Dry. Technol..

[61] Wiktor A., Śledź M., Nowacka M., Chudoba T., Witrowa-Rajchert D. (2014). Pulsed Electric Field Pretreatment for Osmotic Dehydration of Apple Tissue: Experimental and Mathematical Modeling Studies. Dry. Technol..

[62] Ade-Omowaye B., Rastogi N.K., Angersbach A., Knorr D. (2002). Osmotic dehydration of bell peppers: Influence of high intensity electric field pulses and elevated temperature treatment. J. Food Eng..

[63] Taiwo K.A., Angersbach A., Knorr D. (2003). Effects of Pulsed Electric Field on Quality Factors and Mass Transfer During Osmotic Dehydration of Apples. J. Food Process Eng..

[64] Delgado A., Rubiolo A. (2005). Microstructural changes in strawberry after freezing and thawing processes. LWT.

[65] Khuwijitjaru P., Somkane S., Nakagawa K., Mahayothee B. (2022). Osmotic Dehydration, Drying Kinetics, and Quality Attributes of Osmotic Hot Air-Dried Mango as Affected by Initial Frozen Storage. Foods.

[66] Reeve R.M. (1970). Relationships of Histological Structure to Texture of Fresh and Processed Fruits and Vegetables. J. Texture Stud..

[67] Ando Y., Maeda Y., Mizutani K., Wakatsuki N., Hagiwara S., Nabetani H. (2016). mpact of blanching and freeze-thaw pretreatment on drying rate of carrot roots in relation to changes in cell membrane function and cell wall structure. LWT.

[68] Arevalo P., Ngadi M.O., Bazhali M.I., Raghavan G.S.V. (2004). Impact of Pulsed Electric Fields on the Dehydration and Physical Properties of Apple and Potato Slices. Dry. Technol..

[69] Bazhal M.I., Ngadi M.O., Raghavan V.G. (2003). Influence of Pulsed Electroplasmolysis on the Porous Structure of Apple Tissue. Biosyst. Eng..

[70] Filipović V., Filipović J., Lončar B., Knežević V., Nićetin M., Filipović I. (2022). Synergetic dehydration method of osmotic treatment in molasses and successive lyophilization of peaches. J. Food Process. Preserv..

[71] Rupasinghe H.P., Joshi A.P.K., Pitts N.L. (2010). Non-Fried Apple Food Products and Processes for Their Preparation. https://patentimages.storage.googleapis.com/72/8a/aa/4c2c7578a442ec/US20100159082A1.pdf.

[72] Chauhan O.P., Singh A., Singh A., Raju P.S., Bawa A.S. (2011). Effects of Osmotic Agents on Colour, Textural, Structural, Thermal, and Sensory Properties of Apple Slices. Int. J. Food Prop..

[73] El-Aouar Â.A., Azoubel P.M., Barbosa J.L., Xidieh Murr F.E. (2006). Influence of the osmotic agent on the osmotic dehydration of papaya (*Carica papaya* L.). J. Food Eng..

[74] Charoenrein S., Owcharoen K. (2016). Effect of freezing rates and freeze-thaw cycles on the texture, microstructure and pectic substances of mango. Int. Food Res. J..

[75] Dermesonlouoglou E., Chalkia A., Dimopoulos G., Taoukis P. (2018). Combined effect of pulsed electric field and osmotic dehydration pre-treatments on mass transfer and quality of air dried goji berry. Innov. Food Sci. Emerg. Technol..

[76] Bomben J.L., King C.J. (1982). Heat and mass transport in the freezing of apple tissue. Int. J. Food Sci. Technol..

